# An Up-to-Date Review of Natural Nanoparticles for Cancer Management

**DOI:** 10.3390/pharmaceutics14010018

**Published:** 2021-12-22

**Authors:** Daniel Ion, Adelina-Gabriela Niculescu, Dan Nicolae Păduraru, Octavian Andronic, Florentina Mușat, Alexandru Mihai Grumezescu, Alexandra Bolocan

**Affiliations:** 1 General Surgery Department, Faculty of Medicine, Carol Davila University of Medicine and Pharmacy, 050474 Bucharest, Romania; dr.daniel.ion@gmail.com (D.I.); dan.paduraru.nicolae@gmail.com (D.N.P.); andronicoctavian@gmail.com (O.A.); flori.musat94@gmail.com (F.M.); bolocan.alexa@gmail.com (A.B.); 2 3rd Clinic of General and Emergency Surgery, University Emergency Hospital of Bucharest, 050098 Bucharest, Romania; 3 Department of Science and Engineering of Oxide Materials and Nanomaterials, Faculty of Applied Chemistry and Materials Science, Politehnica University of Bucharest, 011061 Bucharest, Romania; adelina.niculescu@upb.ro; 4 Research Institute of the University of Bucharest—ICUB, University of Bucharest, 050657 Bucharest, Romania; 5 Academy of Romanian Scientists, Ilfov No. 3, 050044 Bucharest, Romania

**Keywords:** natural nanoparticles, natural anticancer compounds, natural cancer therapies, novel cancer treatment alternatives, natural nanocarriers, chemotherapeutic agents targeted delivery

## Abstract

Cancer represents one of the leading causes of morbidity and mortality worldwide, imposing an urgent need to develop more efficient treatment alternatives. In this respect, much attention has been drawn from conventional cancer treatments to more modern approaches, such as the use of nanotechnology. Extensive research has been done for designing innovative nanoparticles able to specifically target tumor cells and ensure the controlled release of anticancer agents. To avoid the potential toxicity of synthetic materials, natural nanoparticles started to attract increasing scientific interest. In this context, this paper aims to review the most important natural nanoparticles used as active ingredients (e.g., polyphenols, polysaccharides, proteins, and sterol-like compounds) or as carriers (e.g., proteins, polysaccharides, viral nanoparticles, and exosomes) of various anticancer moieties, focusing on their recent applications in treating diverse malignancies.

## 1. Introduction

Cancer has long been a critical threat worldwide, imposing a global health and economic burden. Cancer cells can evade the immune system, multiply indefinitely, and perform angiogenesis, leading to challenging malignancies that directly damage human life [1,2,3,4,5,6]. 

The most frequently employed treatment option in fighting cancer is chemotherapy, which can be used either alone or in combinatorial approaches with radiotherapy, surgery, or adjuvant therapies (e.g., immunotherapy, hormone therapy, photothermal therapy, photodynamic therapy, and ablative techniques) to produce effective responses depending on the cancer stage [7,8,9,10]. However, the dissatisfying specificity coupled with poor aqueous solubility and short blood circulation of conventional anticancer drugs leads to low concentrations of drugs at the tumor site and the requirement of high doses [2,8,11,12,13]. In addition, the therapeutic efficacy of administered drugs diminishes over time due to the development of drug resistance [14,15]. Despite the growing number of anticancer agents developed in the last few decades, their severe toxicity, high production cost, and low patient compliance demand better antitumor alternatives [16].

**Figure 1:**
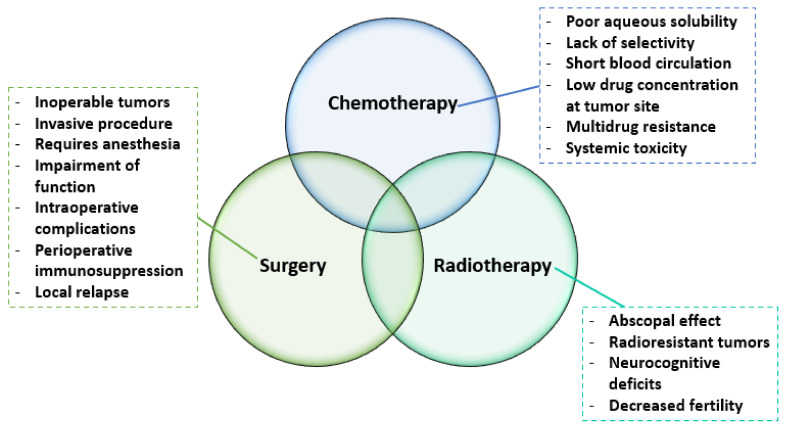
Limitations of conventional cancer treatment strategies. Created based on information from [13,20,21,22,23,24,25].

To improve treatment outcomes, radiotherapy can be used complementarily to destroy cancer cells sensitized by chemotherapeutics. However, radiation also affects normal tissues, leading to side effects occurrence immediately or soon after radiotherapy treatment [17,18]. Thus, the lack of specificity of conventional therapies results in negative effects upon rapidly multiplying normal cells (e.g., bone marrow, gastrointestinal tract, and hair follicles) [13] or other healthy tissues, leading to multiple off-target adverse effects, including appetite loss, anemia, internal bleeding, fatigue, and hair loss [2,12,19]. Some of the most important disadvantages associated with the classic trio of cancer therapeutic options are summarized in Figure 1. 

Nanotechnology has appeared as a promising solution to overcome the most pressing challenges of current cancer treatments [2,26,27]. The use of nanocarriers is a viable option for enhancing solubility and bioavailability of anticancer compounds of both natural and synthetic origin [28], delivering drugs across traditional biological barriers in the body [29], and combining therapeutic agents with imaging techniques towards achieving synergic results [12]. In more detail, anticancer drug delivery via nanoparticles (NPs) is influenced by biological barriers, counting tumor microenvironment (TME) and vasculature, reticuloendothelial system, blood–brain barrier (BBB), and kidney filtration [30]. Thus, special attention must be given to overcoming these barriers and ensuring tumor uptake of NPs.

**Figure 2:**
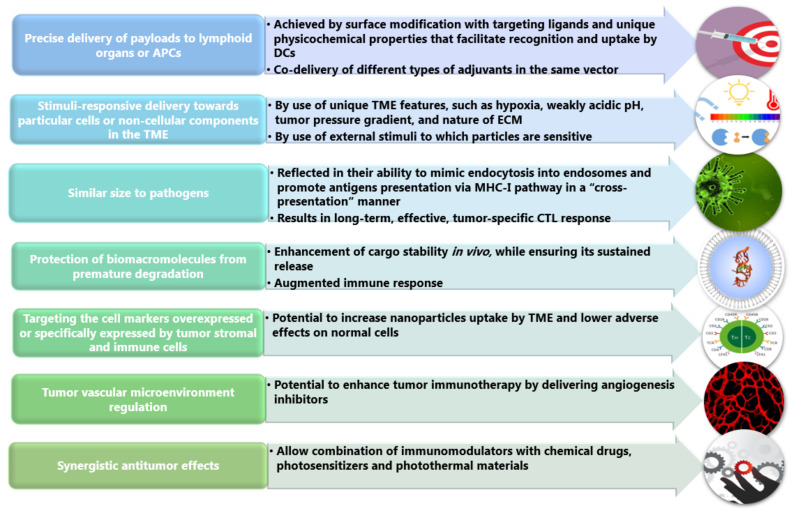
NPs roles in regulating TME and improving tumor immunotherapy. Created based on information from [1,30,34,35,36]. Abbreviations: APCs—antigen-presenting cells; DCs—dendritic cells; TME—tumor microenvironment; ECM—extracellular matrix; MHC—major histocompatibility complex; CTL—cytotoxic T lymphocyte.

Specifically, by taking advantage of the newly accumulated knowledge TME [15,31,32], multifunctional NPs can be designed to deliver bioactive agents directly to the tumor, reducing systemic side effects (Figure 2). This can be done either by surfaced functionalization with ligands specific for receptors expressed by tumor cells or other cells in TME or by functionalization with chemical groups that can respond to TME signals (e.g., secreted molecules, acidic pH, and hypoxic conditions) [11,29,33].

To date, a wide array of materials have been investigated for producing effective anticancer NPs. Researchers developed different nanostructures of different shapes, sizes, architectures, and compositions using various materials, including lipids, proteins, polysaccharides, synthetic polymers, and inorganic materials [2,8,9,11,29]. 

In particular, the beneficial physicochemical and biological properties of natural materials have recently rendered these NPs among the most promising base materials for cancer therapy. As compared to most synthetic NPs, nanostructures of natural origin have been noticed to have better safety profiles, enhanced biocompatibility, biodegradability, and non-immunogenicity; they also present functional groups that facilitate their chemical modification towards obtaining even more performant formulations [8,11,37]. Moreover, as detailed toxicology assessments are fundamental for the clinical translation of nanoparticulate nanomedicines [38], the favorable biological behavior of natural NPs may represent an opportunity for their faster introduction into clinical trials and consequently into medical practice as compared to synthetic alternatives. 

In this respect, the present paper further discusses the most recent advances in natural NPs for cancer management, including both natural molecules with inherent anticancer properties loaded into NPs and natural NPs used as carriers of various freights in cancer therapies. More specifically, in this review are considered “natural” the compounds and materials that can be obtained from biological sources. 

## 2. Natural Compound-Based NPs with Intrinsic Anticancer Activity

Attempting to avoid the side effects and downsides of chemotherapeutic drugs, researchers have tackled the anticancer potential of natural anticancer agents from a variety of plants and organisms [39,40]. Some of the most relevant examples of nanoparticles of natural origin with intrinsic anticancer activity are further described in this section.

### 2.1. Polyphenols

Polyphenols are well recognized for their health benefits, showing biomedical potential in various diseases, such as tumors, inflammatory diseases, and cardiovascular diseases [41,42]. They can be easily included in the daily diet, as they can be found in diverse natural sources (e.g., tea, red wine, cocoa, fruits, and olive oil), or they can be extracted and processed for developing nutraceutical and pharmaceutical formulations with specific and enhanced activity [43].

**Figure 3:**
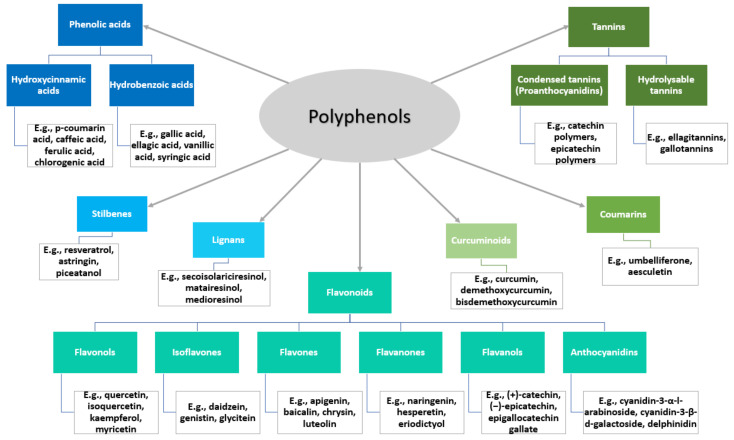
Polyphenols classification and examples. Created based on information from [44,45,46].

Polyphenols represent a broad class of bioactive compounds, comprising a variety of chemical structures (Figure 3). Their molecular structures allow polyphenols to combine with other materials, such as proteins, metal ions, polymers, and nucleic acids, creating better delivery strategies [41]. In this respect, the following subsections discuss the newest approaches for polyphenols delivery that are relevant for alternative or complementary cancer therapies. 

#### 2.1.1. Flavonoids

Nanoparticles obtained from flavonoids have promising anticancer effects [28,47]. One such useful compound is chrysin, which has proven advantageous characteristics, including anti-inflammatory, antioxidant, antiallergic, and cancer chemopreventive properties [2,48,49]. Nonetheless, the poor water solubility and bioavailability of chrysin limit its use as an anticancer drug [48,50]. In this context, special attention has been drawn to investigate chrysin in nanoparticulate form. Mutha et al. [49] have developed chrysin NPs by probe sonication technique. The authors used small amounts of sodium dodecyl sulfate to enhance aqueous solubility of NPs and mannitol as a bulking agent. The as-such obtained NPs demonstrated potential cytotoxicity and significant control on the growth of MCF-7 breast cancer cell line. Another convenient strategy to overcome pure chrysin limitations is to encapsulate this compound in PLGA-PEG nanoparticles [48,50]. For instance, Mohammadian et al. [48] have evaluated chrysin-loaded PLGA-PEG NPs against a gastric cancer cell line. The researchers observed a significant decrease in IC_50_ value of nanocapsulated chrysin as compared to free flavonoid and a decline in miR-18a, miR-21, and miR-221 gene expression. Another example is offered by Tavakoli et al. [50], who have used PLGA-PEG NPs to co-deliver chrysin and curcumin. The scientists reported enhanced antiproliferative and anti-metastatic effects on melanoma cancer when encapsulating these polyphenols than when using them in free form. 

Quercetin is another flavonoid with potential applications in cancer therapy as it exhibits a strong inhibitory effect on the growth of several cancer cell lines, including nasopharyngeal, lung, prostate, ovarian, breast, leukemic, skin, bone, and colon cancer cells [51,52,53]. Nonetheless, the application of quercetin in anticancer treatments is limited by low aqueous solubility, bioavailability, and chemical instability in neutral and alkaline media [54]. To overcome these drawbacks, quercetin can be delivered via different nanoplatforms. For instance, Rezaei-Sadabady et al. [55] have encapsulated quercetin into liposomes to enhance the hydrophilicity and deliverability of this flavonoid. The authors obtained promising results in terms of quercetin solubility and bioavailability, but the types of cancer most likely to benefit from this non-toxic therapy were yet to be determined. Another delivery possibility is proposed by Sadhukhan et al. [56], who have loaded quercetin into phenylboronic acid conjugated zinc oxide NPs. The scientists observed that their nanosystem was able to enhance oxidative stress and mitochondrial damage, leading to apoptotic cell death in human breast cancer cells. Moreover, tumor-associated toxicity in the liver, kidney, and spleen was reportedly reduced. Quercetin was also prepared in combination with other nanomaterials, including chitosan NPs [57,58], PEGylated-PLGA nanocapsules [59], pluronic-grafted gelatin copolymers [60], mesoporous silica NPs [61], and metal–organic frameworks [54]. 

**Figure 4:**
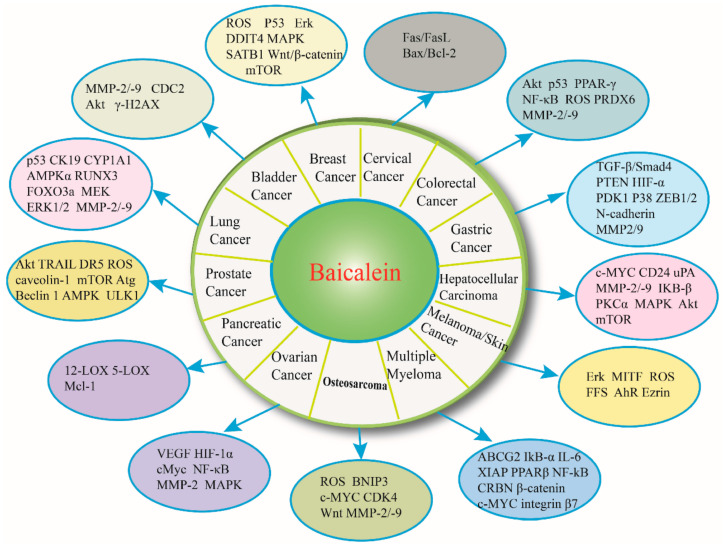
Baicalein anticancer activity by binding to and interacting with specific cellular targets. Reprinted from an open-access source [63].

Baicalein has also been extensively studied as an alternative to synthetic chemotherapeutic agents [2]. It has been proven as an anti-inflammatory, antioxidant, and antitumor agent for different types of cancers, including lung, breast, skin, and gastric cancers [62,63] (Figure 4). To overcome its poor bioavailability caused by its hydrophobic nature, scientists started to explore various baicalein-based nanoparticulate combinations. For instance, Wang et al. [62] developed self-assembled NPs containing dual-targeted ligands of folate and hyaluronic acid for co-delivery of baicalein and paclitaxel. The as-described system was proven efficient for targeted drug delivery, leading to synergistic anticancer effects and overcoming multidrug resistance in human lung cancer cells. A different strategy is offered by Joshi et al. [64], who have fabricated solid lipid nanoparticles of baicalein that had an increase of over ~300% in relative oral bioavailability as compared to free flavonoid administration. The researchers also reported better radioprotection to healthy cells and sensitized cancer cells to radiation-induced killing, concluding that these novel nanoparticles can be employed as an adjuvant in cancer radiotherapy. 

**Figure 5:**
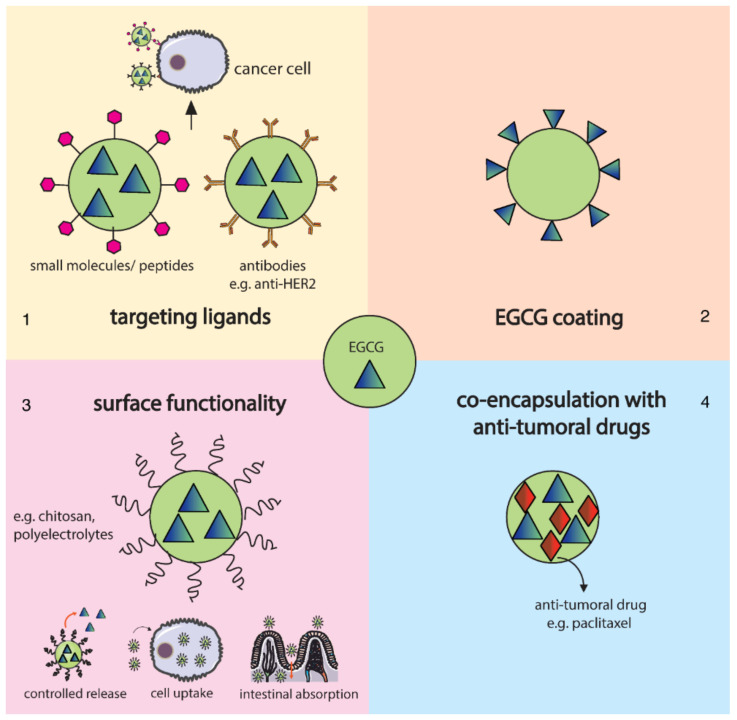
EGCG delivery possibilities for cancer therapy: (**1**) incorporation of ligands on NPs surface for specific targeting of cancer cell receptors or antigens; (**2**) EGCG used as a capping agent; (**3**) surface functionalization with polymers for improving drug release, cellular uptake, and intestinal absorption; (**4**) co-encapsulation with conventional chemotherapeutic agents. Reprinted from an open-access source [66].

Green tea catechins were also noted for their suppressive effects on cancer cell progression, metastasis, and angiogenesis [65]. The major bioactive constituent of green tea, (−)-epigallocatechin-3-gallate (EGCG), has been intensively studied for its chemopreventive and chemotherapeutic activity. However, its lack of target specificity, short half-life, low stability, and low bioavailability limit its free use and request for special delivery approaches [66,67] (Figure 5). EGCG-based nanosystems showed promising synergistic results by conjugation with gold NPs [68,69,70], encapsulation in synthetic [71,72,73] and natural [74,75,76,77] polymeric NPs, liposome delivery [78,79,80,81], and combination with various other anticancer agents [79,81,82,83].

#### 2.1.2. Tannins

Condensed tannin extracts have also been remarked as promising anticancer candidates due to their antitumor activity and potential of inducing apoptosis in cancer cells via enzymes regulation [37,84,85]. Other anticancer mechanisms of tannins include negative regulation of transcription factors, growth factors, receptor kinases, and various oncogenic molecules [86].

Recently, scientists have moved from condensed tannins extracts to their nanoparticles’ counterparts, as is the case of AlMalki et al. [37], who have synthesized NPs from a commercial product extracted from the bark of Pine trees. The researchers obtained potential anticancer effects against MCF-7 cells, concluding that tannin nanoparticles are promising candidates for treating breast cancer either alone or in combination with low doses of tamoxifen. 

#### 2.1.3. Resveratrol

Resveratrol is the most studied stilbene due to its large availability, antioxidant properties, and potential induction of cancer chemopreventive and therapeutic responses [87,88]. Nonetheless, similar to other polyphenols, resveratrol’s direct use is hindered by its low bioavailability and rapid metabolism [88]. 

Thus, the development of nanoparticulated formulations has become a convenient solution for overcoming these drawbacks. Conjugating resveratrol to gold NPs was seen to improve its bioavailability, leading to optimal cellular uptake, and enhanced antitumor efficacy against breast, prostate, and pancreatic cancer cells [89]. Other studies emphasized the potent anticancer activity of resveratrol (RES) when loaded in solid lipid NPs (SLNs [90], functionalized mesoporous silica NPs (MSNs) [91], gelatin NPs [92,93], and more (Table 1).

#### 2.1.4. Curcumin

**Figure 6:**
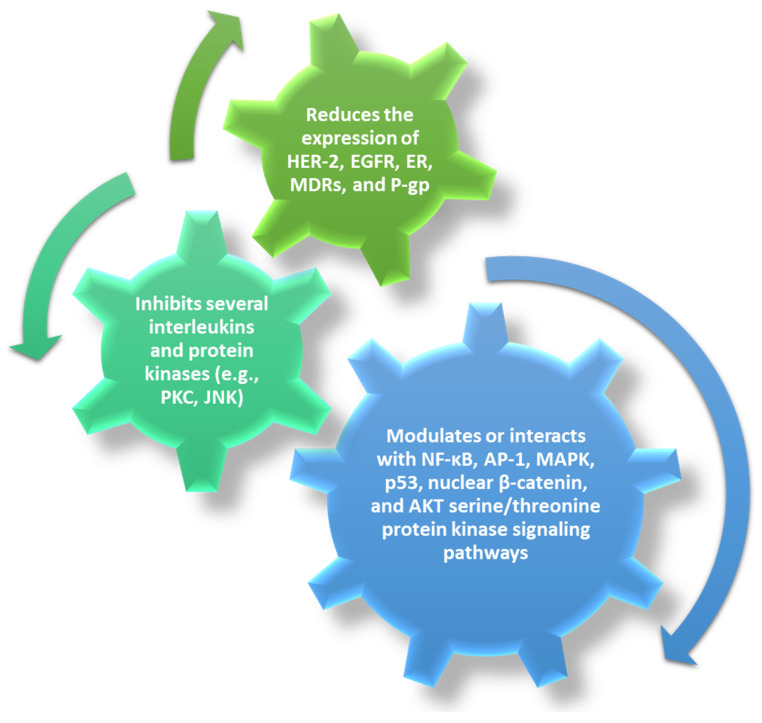
Anticancer effects of curcumin. Created based on information from [39,97].

Curcumin is one of the most studied herbal anticancer compounds, especially due to its multiple-fold action (Figure 6). Curcumin can inhibit carcinogenesis, angiogenesis, and tumor growth [97] by modulating or interacting with growth factors, interleukins, and protein kinases, thus being a promising alternative to conventional chemotherapeutic agents [39,40]. 

However, curcumin has low water solubility and is unstable in physiological conditions, drawbacks that further lead to poor bioavailability and unfavorable biodistribution [39,98]. To overcome these challenges and enhance anticancer bioactivity, scientists employed curcumin (CUR) in the development of composite nanoparticles by conjugation with human serum albumin (HSA) [99], encapsulation in chitosan [40,100], silk fibroin [101], soybean polysaccharide [102], and more (Table 2).

### 2.2. Proteins

Increasing interest has been directed towards using ovalbumin (OVA) protein antigen in developing novel anticancer nanoparticles. This non-toxic, temperature- and pH-sensitive, and economical material is convenient for use in cancer immunotherapy as it elicits cellular and humoral immune responses [108,109]. Habibi et al. [108] have created different OVA-based NPs by chemically linking individual OVA molecules via PEG units. The scientists noticed that by controlling the PEG/OVA ratio, the physicochemical characteristics of the protein NPs, such as size, elasticity, and network structure, can be tailored. The authors reported better results as the PEG/OVA ratio decreased, i.e., increased uptake by lymph node macrophages, dendritic cells, and B cells, more effective processing by dendritic cells, enhanced lymphatic drainage, higher anti-OVA antibody titers in vivo, and overall improved humoral responses. Moreover, when compared to solute OVA, NPs significantly increased the median survival rate in a mouse model of B16F10-OVA melanoma.

Silk sericin is another protein of interest for improving cancer treatment strategies. Sericin’s biocompatibility, non-immunogenicity, and antioxidant properties are the main factors contributing to its research for anticancer purposes. It has been proven that this natural material can reduce oxidative stress or suppress cancer cytokines for skin and colon cancer. Moreover, silk sericin has a self-assembling capacity and unique chemistry that favors surface modifications, thus also being a promising nanocarrier for anticancer drugs [110,111]. 

Keratin may also be employed in designing NPs for cancer therapies. This protein is particularly appealing due to its unique amino acid sequences that can specifically bind vitronectin integrin receptors overexpressed by several cancer cells [112]. Nonetheless, this inherent anticancer potential has been mostly exploited for delivery purposes, to create stimuli-responsive nanocarriers for chemotherapeutics [112,113,114,115], or to enhance the effects of phototherapies [116,117,118,119].

### 2.3. Polysaccharides

Fucoidan is a marine polysaccharide that can be extracted from different species of brown algae. As this polymer exhibited great promise in treating several types of cancer, including colon, bladder, liver, lymphoma, and gastric cancers, researchers started to investigate its effects in the nanoscale form [120,121]. For instance, Etman et al. [120] have developed fucoidan-based NPs by polyelectrolyte interaction with lactoferrin targeted ligand. The researchers have reported cytotoxic properties against pancreatic cancer cells, enhanced ability to prevent tumor cells migration and invasion, with a 2.3-fold decreased IC_50_ value for fucoidan NPs than for fucoidan solution. 

Chitosan, a polymeric biomaterial found in shellfish exoskeletons, can also be used in developing novel anticancer formulations. This polysaccharide manifests its antitumor activity by affecting cell digestion and inhibiting cell development. However, most of the studies approach chitosan as an assistant agent and nanocarrier rather than for its intrinsic anticancer properties [122].

### 2.4. Sterol-Like Compounds

Sterol-like natural compounds have also been shown to have promising antitumor activity, gaining interest in developing innovative nanoparticulate cancer therapeutics. For instance, Qiu et al. [123] have loaded 20(S)-ginsenoside (Rg3) into pH-sensitive polymeric NPs that can target cancer cells and prolong circulation time. It was reported that Rg3 could be released rapidly at the tumor site, significantly inhibiting tumor proliferation. Specifically, the sterol-like compound decreased the expressions of proliferating cell nuclear antigen, producing the apoptosis of colorectal cancer cells through the increased expressions of caspase-3. 

A different approach was used by Kim et al. [124], who have prepared flower-shaped nanocomposites based on zinc oxide and hyaluronic acid, which they functionalized with ginsenoside Rh2. This complex nanosystem proved successful against lung, colon, and breast cancer cells, exerting its anticancer activity through several mechanisms (e.g., ROS production, upregulation of p53 and BAX, downregulation of BCL2, induction of morphological changes in the nucleus of tumor cells). 

## 3. Natural Nanoparticles for Anticancer Drug and Gene Delivery

A lot of research effort has been put into developing biocompatible targeted delivery vehicles from materials of natural origin. Numerous studies have been published evaluating the anticancer action of various natural-based nanosystems able to effectively and efficiently deliver different cargos to the tumor site. Several recently developed nanoformulations based on proteins, polysaccharides, viral NPs, exosomes, and other natural materials are further presented to make an overview of current progress in the field and emphasize the versatility of these nanoparticles for cancer management. 

### 3.1. Protein-Based NPs

#### 3.1.1. Albumin

Human serum albumin (HSA) is an appealing material for developing novel nanomedicines as it has a high drug loading ability, self-assembling properties, long half-life, and preferential uptake by tumor and inflamed tissues [11,125]. Moreover, increasing interest has been drawn to using HSA-based NPs in cancer management after the FDA approval of paclitaxel-bound albumin NPs (Abraxane^TM^) [7,126]. Albumin NPs also show a good affinity for other cancer drugs, such as doxorubicin, curcumin, and tacrolimus [109].

For instance, Chaiwaree et al. [127] have successfully encapsulated doxorubicin in HSA submicron particles. The researchers reported excellent A549 cell uptake (up to 98%) and localization of drug nanoformulation within the cell lysosomal compartment. These observations were also reflected in the reduction of cancer cell metabolic activities after 72 h and less than 1% drug release within 5 h at physiological pH. Thus, it can be concluded that this delivery system is a potential candidate for cancer therapy. 

Another promising strategy was developed by Yu et al. [128], who have fabricated albumin NPs loaded with docetaxel and functionalized with nucleolin-targeted aptamers. Their targeted drug delivery system was preferentially ingested by nucleolin-expressing CT26 colon cancer cells, leading to enhanced antitumor efficacy, low systemic toxicity, and prolonged survival of CT26-bearing mice. 

#### 3.1.2. Keratin

The targeting potential and ease of functionalization of keratin nanoparticles have been exploited by Avancini et al. [116], who have used them as nanocarriers for salinomycin, chlorin e6 photosensitizer, and vitamin E acetate. The researchers tested this novel drug delivery system in vitro on breast cancer cell lines and cancer stem cell (CSC)-enriched mammospheres, reporting synergistic cell killing, limited self-renewal capacity, and eradication of CSCs. Further in vivo tests on zebrafish embryos confirmed the results and revealed that keratin encapsulation of the drug does not alter its CSC-specific cytotoxicity. 

Lu et al. [117] have also investigated keratin-based NPs for photodynamic therapy against breast cancer. The complete nanosystem comprised keratin as nitric oxide generator, phenylboronic acid (PBA)-modified d-α-tocopherol polyethylene glycol 1000 succinate (TPGS) as targeting ligand, and methylene blue as photosensitizer. In vivo studies showed that the developed constructs induced extensive cell apoptosis, leading to significant inhibition of in situ tumor growth and lung metastases. 

#### 3.1.3. Silk Sericin and Fibroin

The two proteins of silk (i.e., sericin and fibroin) can be used for engineering silk-based nanoparticles suitable for drug delivery and cancer treatment [129,130,131]. For instance, Huang et al. [111] have fabricated folate-conjugated sericin nanoparticles for tumor targeting and pH-responsive delivery of doxorubicin. The as-described NPs possessed good cytotoxicity and hemocompatibility, specifically releasing the encapsulated drug freight into the lysosomes of folate receptor-rich KB cells. 

Silk sericin has also found use as a photosensitizer carrier. More specifically, Gao et al. [132] have used this protein to create NPs for chlorin e6 delivery. These nanosystems achieved superior accumulation in tumor sites compared to with free photosensitizer agents, suppressing tumor growth and avoiding side effects occurrence.

Pandey et al. [133] have developed silk fibroin NPs coated with hydrophilic stabilizers to allow longer circulation times and facilitate their uptake by low-density lipoprotein receptors. These nanocarriers loaded with doxorubicin showed a proinflammatory response, sustained drug release, and better cytotoxicity than the free drug, being a potential candidate for glioblastoma treatment.

Silk fibroin was also proven useful in the delivery of drugs for colorectal cancer treatment. Particularly, Hudita et al. [134] have designed silk fibroin-based NPs for the delivery of 5-fluorouracil (5-FU). The scientists reported great antitumor efficacy in vitro and promising results in vivo, as the proposed system was able to ameliorate mucositis induced during 5-FU treatment. 

#### 3.1.4. Ferritin

Ferritin is a convenient protein for drug encapsulation as it self-assemblies naturally into a hollow nanocage with an inner diameter of 7–8 nm. This structure is composed of 24 subunits of either heavy chain ferritin (HFn), light chain ferritin (LFn), or a mix between the two types. In particular, HFn has been noted to bind human cells by interacting with transferrin receptor 1 (TfR1), which is highly expressed on cancer cells and is commonly used as a targeting marker for tumor diagnosis and therapy [135,136]. 

In this respect, Liang et al. [136] have investigated HFn nanocages for the delivery of doxorubicin. The researchers demonstrated that these ferritin nanostructures were able to transport high drug doses for tumor-specific targeting and killing without requiring any additional functionalization or property modulation. Doxorubicin-loaded HFn internalized into tumor cells via interaction with overexpressed TfR1, releasing the drug in lysosomes and significantly inhibiting tumor growth after a single dose injection with minimum healthy organ drug exposure. A similar approach was followed by Jiang et al. [137], who have used doxorubicin-loaded ferritin nanosystems against hepatocellular carcinoma. The scientists also used a targeted ligand (i.e., GRP78-targeted peptide SP94) to improve release selectivity. The as-such designed platform was able to encapsulate a high amount of drug, ensuring a lower dosage of carrier and fewer adverse effects. Moreover, a better therapeutic effect was observed as compared to currently reported nanomedicines.

A different strategy is offered by Sitia et al. [138], who have encapsulated navitoclax and functionalized HFn nanocages with fibroblast activation protein (FAP) antibody fragments aiming to create cancer-associated fibroblast (CAF)-targeted drug delivery agents. The obtained results are promising as there were reported significantly higher drug release only in FAP^+^ cells, and considerably higher cytotoxicity than non-functionalized systems. The researchers concluded it would be interesting to study if this nanoplatform is also able to reduce metastases formation.

### 3.2. Polysaccharide-Based NPs

#### 3.2.1. Chitosan

Chitosan has many advantageous properties recommending it as a suitable delivery vehicle for a variety of biomolecules [139]. Being non-toxic, biodegradable, and biocompatible, chitosan became the material of choice for developing many nanoparticulate formulations for biomedical applications in general, and cancer management in particular [40]. 

For instance, chitosan has been demonstrated successful in improving the efficacy of photodynamic therapy. Ding et al. [140] have prepared chitosan NPs encapsulated with chlorin e6 photosensitizer by a nonsolvent-aided counterion complexation method, noting enhanced biocompatibility and dramatically increased therapy efficiency compared with free photosensitizer administration.

Chitosan NPs have also been remarked as a promising anticancer drug delivery platform [141]. Gounden et al. [142] have developed silver NPs conjugated with chitosan and loaded with cisplatin. The authors reported specificity towards breast cancer cells with minimal cytotoxicity towards normal cells, as the drug was efficiently and rapidly released from the nanosystem at low pH. 

#### 3.2.2. Fucoidan 

Etman et al. [143] have used fucoidan extracted from *Undaria pinnatifida* to encapsulate quinacrine. Fucoidan acted as both delivery vehicle and active targeting ligand, while for some particles lactoferrin was added as a second active targeting ligand. The researchers obtained promising results for both single- and dual-targeting particles against pancreatic cancer, with a higher animal survival rate and no hepatotoxicity. In particular, dual-targeted particles were reported to enhance quinacrine activity 5.7-fold compared to free drug solution, having a higher ability in inhibiting cancer migration and invasion.

Jafari et al. [144] have also taken advantage of fucoidan’s targeting ability. The authors used fucoidan-conjugated doxorubicin nanoparticles to target P-selectin overexpressed by malignant cells, obtaining enhanced cellular uptake and cytotoxicity against the MDA-MB-231 cell line. 

Alternatively, Coutinho et al. [145] have combined fucoidan and chitosan to create an oral delivery system for methotrexate. The nanoplatform was reported safe towards fibroblasts but hindered lung cancer cell proliferation via an apoptotic process, being 7-times more effective than the free drug.

#### 3.2.3. Alginate

Alginate has attracted research interest especially due to its biocompatibility, biodegradability, ease of production, and functionalization [146]. This natural polymer has been used as a base material for several novel anticancer formulations. As an example, Pourjavadi et al. [147] have employed this polysaccharide in the development of multifunctional nanocarriers. The researchers fabricated a magnetic core nanocarrier with a lipophilic surface based on oleic acid chains onto which paclitaxel and doxorubicin were adsorbed and covered by a smart pH-sensitive alginate shell. The system exhibited increased stability, enhanced biocompatibility, faster drug release in the acidic medium than at physiological pH, and even higher toxicity toward MCF-7 and HeLa cells than the free drugs. 

#### 3.2.4. Hyaluronic Acid

Hyaluronic acid (HA) is another polysaccharide [148] of interest for cancer drug delivery especially due to its ability to specifically bind CD44 receptor overexpressed by cancer cells [11]. For instance, Gaio et al. [149] have proposed the co-delivery of docetaxel and meso-tetraphenyl chlorine disulfonate (TPCS_2a_) photosensitizer via HA-coated NPs. Combining chemotherapy and photodynamic therapy, their nanosystem demonstrated superior efficacy over monotherapies in lowering self-renewal capacity and inhibiting the growth of breast cancer CSCs.

Moreover, thiolated HA (HA-SS) is sensitive to glutathione [19], and is thus a potential targeting agent for TME-responsive delivery. In this respect, Debele et al. [150] have created a dual-sensitive HA-SS conjugated with 6-mercaptopurine for doxorubicin-targeted delivery to parental colon cancer and colon cancer CSCs. The synthesized nanoformulation was uptaken via CD44 receptor, accumulating more in the tumor region than in any other organ. 

### 3.3. Viral NPs

The use of plant viruses in humans is considered a safe and promising alternative for intravital imaging and drug delivery [151]. Particularly, cowpea mosaic virus (CPMV) was reported advantageous due to its capsid’s icosahedral shape, which allows enhanced multifunctional group display and endows the viral NP with the ability to carry specific cargos. In what concerns cancer therapies, CPMV was noted to have enhanced permeability and retention effect, allowing these viral NPs to preferentially extravasate from tumor neovasculature and efficiently penetrate tumors [152]. 

For instance, Lam et al. [153] have used CPMV for the delivery of mitoxantrone (MTO) antineoplastic chemotherapeutic to treat glioblastoma multiforme (GBM). The researchers reported CPMV-MTO uptake in glioma cells and significant in vitro cytotoxic effects both as a solo therapy and in combination with tumor necrosis factor-related apoptosis-inducing ligand, concluding that these plant virus-based NPs are promising platforms for GBM treatment. 

Cowpea chlorotic mottle virus (CCMV) has also been exploited in developing innovative cancer treatments. Cai et al. [154] have employed this plant virus in the targeted delivery of oligodeoxynucleotides (ODN) with CpG motifs to tumor-associated macrophages (TAMs). This nanoformulation promoted ODN uptake by TAMS, enhancing their phagocytic activity. Moreover, the direct injection of these engineered NPs into tumor tissues induced a robust antitumor response increasing the phagocytic activity in the TME. 

Another plant-based virus reported for anticancer drug delivery is tobacco mosaic virus (TMV). Franke et al. [155] used TMV as a nanocarrier of cisplatin as a potential treatment for platinum-resistant ovarian cancer. The scientists observed more efficient cell uptake, superior cytotoxicity and DNA double-strand breakage in both platinum-sensitive and platinum-resistant cancer cells than for free cisplatin, concluding that their newly developed nanoplatform may be a powerful tool in combating ovarian cancer. 

Other viruses reported for cancer applications include potato virus X, red clover necrotic mosaic virus, papaya mosaic virus and physalis mottle virus [151].

### 3.4. Exosomes

Exosomes have recently emerged as potential nanocarriers of anticancer therapeutics due to their biocompatibility, low immunogenicity, long circulation time, and high loading capacity. These nanoscale extracellular vesicles benefit from an excellent tumor cell uptake and high specificity to tumor-associated cells [19,29]. 

Aqil et al. [156] have used bovine milk exosomes as nanocarriers for siRNA delivery. The scientists reported a dose-dependent antiproliferative activity against A549 cells treated with folic acid-functionalized exosomes loaded with siKRAS^G12S^ gene, concluding that this nanocarrier is suitable for siRNA delivery and effective for tumor growth inhibition. 

Another application of bovine milk exosomes is offered by Li et al. [157], who have encapsulated doxorubicin in these vesicles and decorated them with HA as a targeting ligand for the selective delivery into overexpressed CD44 tumor cells. Munagala et al. [158] have also developed drug-loaded exosomes for cancer therapy. The authors loaded various chemopreventive and chemotherapeutic agents (i.e., withaferin A, bilberry-derived anthocyanidins, curcumin, paclitaxel, and docetaxel) into exosomes and used folic acid to achieve tumor targeting, reporting enhanced biological efficacy, improved specificity, and elimination of off-target side effects of encapsulated drugs. 

Furthermore, cancer cell-derived exosomes have unique characteristics that can be exploited in early cancer diagnosis, detecting highly metastatic cancer cells, and assessing cancer heterogeneity [159].

### 3.5. Other Natural NPs

Studies have also evaluated drug delivery nanoplatforms that do not fit under any of the above-presented categories. One such example is proposed by Carvalho et al. [160], who have encapsulated *Solanum lycocarpum* alkaloidic extract in natural lipid-based NPs with the aim of creating a better bladder cancer treatment strategy. The authors reported a sustained release profile 36 h after administration, antitumor activity in targeted cancer cells, and high antiproliferative activity, with a 5.4 times lower cell viability than with free extract. 

Olive oil nanocapsules have also been described in the literature as efficient delivery vehicles for anticancer drugs. For instance, Galisteo-Gonzalez et al. [161] have developed nanocarriers with an olive oil core covered by a cross-linked HSA shell loaded with curcumin. The as described nanoplatforms had a similar IC_50_ value to that of free curcumin, but also avoided issues associated with excipients and displayed an excellent uptake performance (entered human breast cancer cells massively in less than one minute). Another approach of using olive oil nanocapsules was taken by Navarro-Marchal et al. [162], who have surrounded them with a different shell containing phospholipids, a nonionic surfactant and deoxycholic acid molecules, further coated with an αCD44 antibody. This complex nanosystem was loaded with paclitaxel, leading to high targeted uptake and increased antitumor efficacy (up to four times compared to free drug in pancreatic CSCs).

As it has been recently suggested that hydroxyapatite NPs have selective anticancer activity for lung cancer cells, this natural inorganic material can also be employed in designing targeted delivery vehicles [163]. Thus, Chen et al. [164] have developed hafnium-doped hydroxyapatite NPs able to enhance photodynamic therapy efficiency and tumor growth when bombarded with ionizing radiation. The authors concluded that their newly developed nanoplatform is suitable for palliative treatment after lung surgical intervention. 

Recent progress has been reported in developing DNA nanocages for delivery purposes. For instance, Tam et al. [113] have designed self-assembled DNA nanocages functionalized with or without blood–brain barrier (BBB)-targeting ligands. Their nanocarriers were able to carry anticancer drugs and penetrate BBB to inhibit the tumor growth in a U-87 MG xenograft mouse model, being a safe and cost-effective targeted delivery platform for brain tumors. DNA nanocages were also proposed as multifunctional vehicles, such as platforms for the co-delivery of anti-miR-21 and doxorubicin [165], or fluorometric detection of human 8-oxoG DNA glycosylase 1 and doxorubicin delivery [166].

Another highly researched recent delivery concept in cancer therapy is the use of bio-inspired nanoparticles mimicking natural components in the body [7,167]. More specifically, cell membrane coating technology has emerged as a promising strategy to deliver drugs into tumors as it endows nanomaterials with functions and properties that are inherent to source cells [29,168]. In this respect, researchers started to disguise nanoparticles using the plasma membranes of various cell types, including erythrocytes [168,169,170,171], leukocytes [172,173], platelets [174,175], macrophages [176,177], bacterial cells [178], stem cells [179,180], cancer cells [181,182], and different hybrid cell coatings [183,184,185,186]. 

### 3.6. Summative Discussion

**Figure 7:**
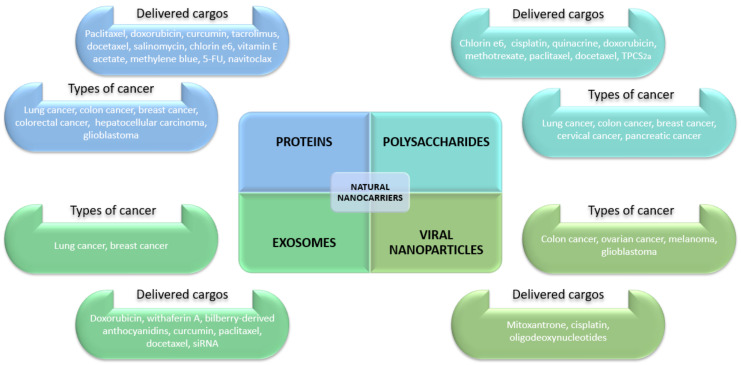
Overview of the main discussed categories of natural nanocarriers for cancer management.

A variety of natural materials can be employed in developing nanoconstructs for safe and efficient anticancer therapies. Incorporating a wide range of either conventional drugs or natural bioactive molecules, natural nanocarriers have been studied against diverse cancer cell lines in vitro or as in vivo therapeutic approaches in animal models. Summarizing the above-discussed research studies, Figure 7 overviews the four main categories of nanocarriers in a clear and concise manner in relation to their delivered moieties and cancer types for which they have been investigated. 

Proteins and polysaccharides can be considered dual-role biomaterials as some of them can act both as delivery vehicles and active ingredients. Moreover, several exponents of these categories may be concomitantly used as active targeting ligands (e.g., fucoidan, hyaluronic acid, ferritin, and keratin). Nonetheless, additional research is needed for improving the yields of synthesis methods, reducing inter-batch variability, and reducing the costs implied by the design of precisely tailored NPs.

Viral NPs represent an interesting alternative as the use of plant viruses is considered harmless for drug delivery in humans. Moreover, these nanoplatforms have been reported successful against drug-resistant tumors. Nevertheless, their in vivo behavior is still difficult to predict, demanding an individual investigation of each viral-based delivery system.

Exosomes and biomimetic NPs have recently gained interest in the scientific world, being highly promising natural carriers. However, these nanostructures are still in their infancy in cancer management, requiring more research, especially concerning optimizing their preparation processes. 

In addition, comparative data on the performance and treatment efficiency of various natural-origin-based NPs are still scarce in the literature [187]. In this context, further research should also be focused on testing several types of nanocarriers in the same experimental conditions in order to create a basis of understanding of which is the best material and design option for a given type of tumor. 

In an attempt to compare at least from a qualitative point of view the discussed biomaterials, Table 3 summarizes the advantages and disadvantages of each class of the presented nanocarriers. 

Moreover, natural NPs are considered attractive for developing theranostic systems. Through an interdisciplinary approach, multifunctional and multivalent nanostructures can be created towards generating innovative and performant cancer therapies [7,152]. Specifically, several of the discussed nanocarriers have been reported able to encapsulate and co-deliver imaging moieties, drugs, and genes, and even detect cancer cells by binding to specific receptors. Thus, complex natural-based nanoparticulated formulations may hold the answer for effective cancer management. 

## 4. Conclusions

To summarize, cancers remain a global health concern, requiring urgently improved therapeutic strategies. As the current treatment options are limited by invasiveness, severe adverse effects, and poor patient compliance, increasing scientific interest has been shifted to developing carrier systems able to ensure targeted delivery and controlled drug release. Numerous studies have revolved around various synthetic and natural nanomaterials as delivery platforms, yet, in recent years, the latter started to gain more ground. Alternatively, several natural anticancer compounds have been reported to be fabricated into nanoparticles, eliminating the need for synthetic chemotherapeutic agents. 

Thus, through an interdisciplinary approach, better treatment strategies can be envisaged in the foreseeable future. Nonetheless, before applying the described nanoformulations in the clinic, more detailed research has to be performed concerning their efficacy and safety in human use. Moreover, further progress in drug encapsulation optimization, ligand conjugation efficiency, and high reproducibility fabrication with low costs is needed prior to undergoing mass production.

## Figures and Tables

**Table 1:** Examples of anticancer nanoparticle formulation based on resveratrol.

Nanoformulation	Physicochemical Characteristics	Type(s) of Cancer	Observations	Refs.
RES-conjugated gold NPs	Shape: spherical Average core size: 16.1 ± 5.0 nm Zeta potential: −25 mV	Breast cancer Prostate cancer Pancreatic cancer	Enhanced bioavailability of RES Excellent loading of RES with subsequent efficient antitumor effects Synergic anticancer activity due to dual action of gold and RES	[89]
RES-loaded SLNs	Shape: spherical Average diameter: 168 ± 10.7 nm Zeta potential: −23.5 ± 1.6 mV Loading capacity: 25.2 ± 1.7%	Breast cancer	Enhanced bioavailability and anticancer activity of RES Cell proliferation inhibited in a dose-dependent manner Lower IC_50_ values for RES-SLNs than for free RES Increased cell cycle arrest in the G0/G1 phase via CyclinD1 downregulation in cancer cells	[90]
Chitosan-coated-trans-RES and ferulic acid loaded SLNs conjugated with folic acid	Shape: spherical Average diameter: 174 ± 5 nm Zeta potential: −25.9 mV	Colon cancer	Good stability under acidic conditions Effectively involved and increased cytotoxicity in cancer cells, resulting in apoptosis induction Cancer cells specific delivery; induced cell death of HT-29 cell line but did not affect normal NIH 3T3 cells	[94]
RES-loaded in functionalized MSNs	Shape: spherical Average diameter: ~60 nm	Prostate cancer	Significant control over RES release at 5.5 pH Robust and dose-dependent sensitization of Docatexal in hypoxic cell environment Enhanced antiproliferative potential	[91]
RES-loaded PLGA NPs	Shape: spherical Average diameter: 237.8 ± 4.93 nm Encapsulation efficiency: 89.32 ± 3.51%	Prostate cancer	Significant decrease cell viability with IC_50_ and IC_90_ of 15.6 ± 1.49 and 41.1 ± 2.19 μM, respectively Significantly greater cytotoxicity than free RES Anticancer effects mediated by apoptosis; confirmed by cell cycle arrest at G1-S transition phase, DNA nicking, loss of mitochondrial membrane potential, ROS generation, and externalization of phosphtidylserine	[95]
RES-loaded gelatin NPs	Shape: spherical Average diameter: 294 nm Encapsulation efficiency: 93.6%	Non-small cell lung cancer	Superior efficacy in NCI-H460 cells Induced apoptosis via alteration in expression of p53, p21, caspase-3, Bax, Bcl-2 and NF-κB Induced cell arrest in the G0/G1 phase of cell cycle	[93]
RES-cyclodextrin complex-loaded PLGA NPs	Shape: spherical Average diameter: 264.2 ± 3.4 nm	Non-small cell lung cancer	Improved RES aqueous solubility by 66-folds Intensified anticancer effects compared to free RES Enhanced cellular uptake, cytotoxicity, and apoptosis Very good aerosolization potential	[96]

**Table 2:** Examples of anticancer nanoparticle formulations based on curcumin.

Nanoformulation	Physicochemical Characteristics	Type(s) of Cancer	Observations	Refs.
CUR-conjugated with HSA	Shape: spherical Average diameter: 180 ± 2nm Zeta potential: −7mV Loading capacity: 12% Encapsulation efficiency: 70%	Breast cancer	Enhanced stability of CUR both in physiological and acidic conditions Significant increase in CUR cytotoxicity on cancer cells without increasing the toxicity on healthy cells	[99]
CUR and liquid fluorocarbon perfluorohexane (PHF) co-loaded in ferritin nanocages conjugated with folic acid	Shape: spherical Average diameter: 47 nm Zeta potential: −37mV CUR loading ratio: 125.8 ± 2.1%	Ovarian cancer	Significant tumor treatment effects Under low-intensity focused ultrasound (LIFU) and 5.0 pH, the nanoplatform released 53.2% of encapsulated drugs in 24 h After 4 min of LIFU at 5.0 pH, the system provided contrast-enhanced ultrasound imaging capabilities	[103]
CUR-loaded chitosan NPs	Shape: spherical Average diameter: 115 ± 21 nm Zeta potential: 33.8mV Loading capacity: 21.6% Encapsulation efficiency: 83.8%	Colon cancer Lung cancer	CUR was mostly released in the first 5 h then gradually released up to 90 h Higher release in pH 5.2 than in pH 7 Time-dependent decrement of cancer cells viability After 96 h of exposure 67.6% HCT-116 cells and 73.8% A-546 cells were dead	[40]
CUR-loaded chitosan NPs	Shape: spherical Average diameter: 415.30 ± 9.03 nm Zeta potential: 33.37 ± 0.21 mV Encapsulation efficiency: 73.56 ± 6.01%	Lung cancer	Effective and precisely controllable NPs induced cytotoxicity only upon irradiation with 457 nm LED light NPs Upon photoactivation, CUR induced chromatin condensation and DNA fragmentation leading to cancer cells destruction	[100]
CUR-loaded silk fibroin NPs	Shape: spherical Average diameter: 155–175 nm Zeta potential: −45 mV	Hepatocellular carcinoma Neuroblastoma	Local long-term sustained drug delivery Cytotoxicity against cancer cells, while no decreasing viability reported for healthy cells Higher efficacy against neuroblastoma cells than against hepatocellular carcinoma cells	[101]
CUR-loaded soybean polysaccharide nanocapsules	Shape: spherical Average diameter: 200–300 nm Encapsulation efficiency: ~90%	Colon cancer Mammary adenocarcinoma	No significant difference in the viability of HCT 116 and MCF-7 cells challenged with DMSO-dissolved and nanoencapsulated CUR Most of antiproliferative activity of the nanosystem manifested after sim, ulated gastric and intestinal digestions	[102]
CUR-loaded PEGylated MSNs	Shape: spherical Average diameter: 197 nm Loading capacity: 8.1% Encapsulation efficiency: 89.1%	Cervical cancer	Significantly increased solubility and enhanced bioavailability of CUR for photodynamic therapy Smooth and steady release at physiological pH, while at 5.0 pH the release rate was slightly speeded up	[104]
CUR-loaded poloxamer188-*b*-PCL NPs	Shape: spherical Average diameter: 100 nm	Esophageal squamous carcinoma	Improved in vitro antioxidant activity compared to crude CUR powder Particles could biodistribute into liver, kidney, and lung tissues, acting as protection agents in cancer radiotherapy	[105]
CUR-loaded therapeutic lipid NPs	Shape: spherical Average diameter: 19.8 ± 4.2 nm	Nasopharyngeal carcinoma (NPC)	Effective targeting ability, suppressed cellular proliferation, and induced apoptosis in vitro Enhanced inhibitory effect on NPC tumor growth and metastasis in vivo	[106]
CUR-loaded in niosomal NPs	Shape: spherical Average diameter: ~60 nm Zeta potential: −35 mV	Glioblastoma	Dose-dependent decrease in cell proliferation and viability of glioblastoma stem-like cells (GSC) Higher effects on GSC viability, apoptosis, cell cycle arrest, and expression of Bax than free CUR Significantly impaired GSC migration	[107]

**Table 3:** Advantages and disadvantages of main discussed natural nanocarriers for cancer management.

Nanocarrier Type	Examples	Advantages	Disadvantages	Refs
Proteins	Albumin, keratin, silk fibroin, silk sericin, ferritin	Inherent targeting potential Increased cellular uptake Good affinity to anticancer drugs High drug-binding capacity High stability Possibility of self-assembly Ease of functionalization Possibility of use in photodynamic therapy	Batch-to-batch variations Low yields High costs Some may cause in vivo inflammation	[11,109,116,117,132,133,135,136,187,188,189]
Polysaccharides	Chitosan, fucoidan, alginate, hyaluronic acid	Wide availability Ease of production Ease of functionalization Possibility of developing multifunctional carriers Some may act as targeting agents High stability Possibility of use in photodynamic therapy	Complicated manufacturing process Unclear metabolism in the body Poor solubility in common solvents	[11,109,140,143,144,146,147,190,191]
Viral NPs	Cowpea mosaic virus, cowpea chlorotic mosaic virus, tobacco mosaic virus, potato virus X, red clover necrotic mosaic virus, papaya mosaic virus, physalis mottle virus	Enhanced multifunctional group display Some offer the possibility to treat platinum-resistant cancers Possibility of use in photodynamic therapy Can be used for intravital imaging, drug and gene delivery, and immunotherapy	Difficult to predict in vivo behavior; each viral-based delivery system must be evaluated on a case-by-case basis prior to clinical testing	[151,192,193]
Exosomes	Bovine milk exosomes, cancer cell-derived exosomes	High loading capacity for drugs and genes High stability Inherent targeting potential Excellent tumor cell uptake High specificity to tumor-associated cells Enhanced permeability and retention effect Can be employed in cancer diagnosis	Lack of consensus on the best method for obtaining high yields of pure exosomes Challenging to load cargos and targeting agents without corrupting exosomes	[19,29,159,194]
Biomimetic NPs	NPs coated with the membranes of red blood cells, white blood cells, platelets, macrophages, bacterial cells, stem cells, cancer cells	Unique construction Long lifespan Excellent targeting ability Enhanced drug retention Retained cytotoxicity to cancer cells	Some may raise immunogenicity concerns Challenging translation from bench to bedside Complex preparation process Low yields	[29,167,168,195]

## Data Availability

Not applicable.
